# Liposome-Encapsulated Berberine Alleviates Liver Injury in Type 2 Diabetes via Promoting AMPK/mTOR-Mediated Autophagy and Reducing ER Stress: Morphometric and Immunohistochemical Scoring

**DOI:** 10.3390/antiox12061220

**Published:** 2023-06-05

**Authors:** Safaa I. Khater, Taghreed N. Almanaa, Doaa M. Abdel Fattah, Tarek Khamis, Mona M. Seif, Naief Dahran, Leena S. Alqahtani, Mohamed M. M. Metwally, Mahmoud Mostafa, Raghad A. Albedair, Azza I. Helal, Manal Alosaimi, Amany Abdel-Rahman Mohamed

**Affiliations:** 1 Department of Biochemistry, Faculty of Veterinary Medicine, Zagazig University, Zagazig 44511, Egypt; 2 Department of Botany and Microbiology, College of Science, King Saud University, Riyadh 11451, Saudi Arabia; 3 Department of Pharmacology, Faculty of Veterinary Medicine, Zagazig University, Zagazig 44519, Egypt; 4 Laboratory of Biotechnology, Faculty of Veterinary Medicine, Zagazig University, Zagazig 44519, Egypt; 5 Department of Anatomy, Faculty of Medicine, University of Jeddah, Jeddah 80203, Saudi Arabia; 6 Department of Biochemistry, College of Science, University of Jeddah, Jeddah 80203, Saudi Arabia; 7 Department of Pathology, Faculty of Veterinary Medicine, Zagazig University, Zagazig 44511, Egypt; 8 Department of Pharmaceutics, Faculty of Pharmacy, Minia University, Minia 61519, Egypt; 9 Department of Histology and Cell Biology, Faculty of Medicine, Kafrelsheikh University, Kafrelsheikh 33516, Egypt; 10 Department of Basic Medical Sciences, College of Medicine, Princess Nourah Bint Abdulrahman University, Riyadh 11671, Saudi Arabia; 11 Department of Forensic Medicine and Toxicology, Faculty of Veterinary Medicine, Zagazig University, Zagazig 44511, Egypt

**Keywords:** autophagy, AMPK/mTOR, Bclin-1, liposomal berberine, ER stress, type 2 diabetes

## Abstract

In the advanced stages of type 2 diabetes mellitus (T2DM), diabetic liver damage is a common complication that can devastate a patient’s quality of life. The present study investigated the ability of liposomal berberine (Lip-BBR) to aid in ameliorating hepatic damage and steatosis, insulin homeostasis, and regulating lipid metabolism in type 2 diabetes (T2DM) and the possible pathways by which it does so. Liver tissue microarchitectures and immunohistochemical staining were applied during the study. The rats were divided into a control non-diabetic group and four diabetic groups, which are the T2DM, T2DM-Lip-BBR (10 mg/kg b.wt), T2DM-Vildagliptin (Vild) (10 mg/kg b.wt), and T2DM-BBR-Vild (10 mg/kg b.wt + Vild (5 mg/kg b.wt) groups. The findings demonstrated that Lip-BBR treatment could restore liver tissue microarchitectures, reduce steatosis and liver function, and regulate lipid metabolism. Moreover, Lip-BBR treatment promoted autophagy via the activation of LC3-II and Bclin-1 proteins and activated the AMPK/mTOR pathway in the liver tissue of T2DM rats. Lip-BBR also activated the GLP-1 expression, which stimulated insulin biosynthesis. It decreased the endoplasmic reticulum stress by limiting the CHOP, JNK expression, oxidative stress, and inflammation. Collectively, Lip-BBR ameliorated diabetic liver injury in a T2DM rat model with its promotion activity of AMPK/mTOR-mediated autophagy and limiting ER stress.

## 1. Introduction

Ninety-five to one hundred percent of all people with diabetes have type 2 diabetes, often known as T2DM [1]. Dietary and physical activity adjustments, oral hypoglycemic medications, and/or subcutaneous insulin injections are the gold standard for treating type 2 diabetes [2,3]. Oral hypoglycemic agents (OHA) and insulin have come a long way, although there are still shortages. Far from being satisfactory, only 41% of people with diabetes achieve optimal glycemic control with antidiabetic medicines [4]. No therapy could sustain stable blood glucose control for years [5]. Diabetes mellitus is developed only in the case of insulin deficiency, and hyperglycemia results from insulin deficiency. Insulin resistance can be present, but diabetes mellitus can never happen if adequate insulin secretion exists. Long-term management of blood sugar levels requires preventative measures to address pancreatic beta cell failure [6]. Furthermore, type 2 diabetes is a serious health condition that can cause damage to the liver [7]. There is a correlation between type 2 diabetes and fatty liver disease. This can lead to inflammation and scarring of the organ, which can eventually lead to cirrhosis and other complications. Additionally, people with type 2 diabetes may be more likely to develop non-alcoholic steatohepatitis (NASH), a form of fatty liver disease associated with insulin resistance [7]. Maintaining a healthy lifestyle, including regular exercise and a balanced diet, is especially important for persons with type 2 diabetes who want to lower their risk of developing these serious liver problems. As a result, there must be an ongoing quest for safe and effective natural remedies and alternative pharmaceuticals for type 2 diabetes [8].

The isoquinoline alkaloid berberine (BBR) has been employed to treat various conditions, including cancer, diabetes, cardiovascular disease, hypertension, and Alzheimer’s disease, in traditional medical systems such as those in Ayurveda, Iran and China [9]. Wang, et al. [10] stated that berberine’s antidiabetic effect and lipid-lowering effects had been verified by Guo, et al. [11]. Many investigations on the effects of BBR on T2DM have shown that it is effective as an antidiabetic and antioxidant [12,13]. Nonetheless, BBR’s low solubility, quick metabolism, and substantial degradation may limit its capacity to reach the tissues in adequate quantities for displaying local antioxidant and anti-inflammatory actions, limiting its utility in managing type 2 diabetes [14]. As a result, nanoparticles (NPs) are increasingly incorporated into food through the engineering of novel dietary formulations, which provide functional food ingredients with a wide range of unique ways to improve bioavailability, quality, and safety, as well as increase stability and preserve active ingredients from intestinal degradation [15]. Berberine-loaded liposomes (Lip-BBR) were prepared by different methods described by ZHANG, et al. [16], including the ethanol injection method [17], and BBR hyaluronate-based liposomes were also prepared by using the film hydration method [18]. Nano liposomes incorporated in BBR are efficient for improving bioactive solubility, thus enhancing bioavailability and stability [19]. Liposomes are biocompatible and safe, and their formulation in oral delivery systems can increase the bioavailability of medications that aren’t very water-soluble. When compared to more traditional solid dosage forms used for oral drug delivery, liposomes as nanotechnology-based drug delivery systems have many advantages [20]. In addition, the release of a loaded medicine can be targeted in a particular site, and liposomes can encapsulate both hydrophilic and hydrophobic substances (in an aqueous-based core or phospholipid bi-layer) [21].

Lip-BBR could be useful in T2DM via promoting insulin secretion [22], reduction of insulin resistance [23], gluconeogenesis [24], glucose uptake activation and glycolysis [25], and reducing inflammation [26]. BBR also could promote numerous important enzymes [27,28] and manage gut microbiome diseases, according to a study by Zhang, et al. [29].

Since autophagy regulates glucose and lipid metabolism and insulin release, it plays a crucial role in regulating T2DM and its consequences. Many other tissues, including the liver and beta cells, are also affected [30]. Autophagy has been shown to increase the likelihood of cell survival by facilitating adaptive responses that protect against or lessen the consequences of ER stress, mitochondrial dysfunction, and oxidative stress [31]. The stress of the ER originates as a response to the presence of too many unfolded proteins, causing an imbalance between a load of proteins entering the secretory pathway and the capacity of the ER to fold and process them, which is a significant source of cellular stress [32]. When ER stress is detected, a series of proteins involved in signal transduction is activated, known as the unfolded protein response (UPR). In contrast, prolonged activation of the UPR triggers multiple apoptotic signaling cascades, including the CCAAT/enhancer-binding homologous protein (CHOP)-mediated apoptotic pathway [33]. Consistent evidence points to ER stress’s role in the etiology of protein misfolding, including metabolic disorders (diabetes, cardiovascular disease, and non-alcoholic fatty liver). Therefore, cytoprotection against ER stress-induced cellular damage requires the development of pharmacological modulators [34]. Conversely, the AMP-activated protein kinase (AMPK) is a key regulator of cellular and whole-body energy balance [35]. For this reason, AMPK has been investigated as a potential therapeutic target for metabolic disorders, including type 2 diabetes. In addition, it has been observed that AMPK activation can decrease ER stress, suggesting that AMPK can be a therapeutic target for treating ER stress-mediated metabolic illness, especially in hepatocellular damage associated with T2DM [36].

However, despite extensive research in the field, there is currently a dearth of published studies investigating the potential beneficial impact of berberine-loaded liposomes (Lip. BBR) as a novel nano-herbal medicine for mitigating hepatic detrimental effects induced by a high-fat diet and type 2 diabetes mellitus (T2DM) in a rat model. Therefore, the primary objective of our research study was to explore the efficacy of Lip. BBR in the treatment of hepatic complications associated with T2DM in rats.

To achieve this goal, we first induced T2DM in the rats, following which rats were administered Lip. BBR orally at a dose of 10 mg/kg body weight. The Lip. BBR treatment was administered either alone or in combination with vildagliptin (VILD), a well-established antidiabetic drug. This approach allowed us to assess the influence of Lip. BBR on various aspects of glucose homeostasis, lipid metabolism, hepatic oxidants/antioxidants status, and liver microarchitectures. Furthermore, we investigated the effects of Lip. BBR on autophagy, endoplasmic reticulum stress, and lipid metabolism based on its previously examined properties [37,38].

In our study, we focused on understanding the role of Lip. BBR as an autophagy modulator and elucidating the associated signaling pathways, including AMPK/mTOR, Beclin-1, and P62, in relation to hepatic tissue protection in the T2DM rat model. Our findings demonstrated that Lip. BBR exerts a regulatory influence on basal autophagy in mature adipocytes, providing a promising strategy for combating obesity and its associated metabolic syndrome. Additionally, we observed that BBR enhances autophagy through AMPK activation while concurrently mitigating high glucose-induced mTOR activation, thereby safeguarding against damage to podocytes caused by high glucose levels [39].

## 2. Materials and Methods

### 2.1. Test Compounds

Berberine chloride and streptozotocin (STZ) were attained from Sigma-Aldrich (Sigma, St. Louis, MO, USA). STZ was mixed in freshly buffered citrate (0.71 g of sodium dibasic phosphate in 100 mL DW) for induction of T2DM. It was titrated against 0.96 g of acetic acid in 100 mL until it reached a pH of 4.5 and injected intraperitoneally (I/P) in rats according to the method of Mohemed et al. [3]. Cayman was the source for our vildagliptin (Cas no. 274901-16-5). Its chemical name is “(2S)-1-[2-[(3-hydroxy-1-adamantyl) amino” and it is an acetyl chemical compound with the IUPAC name pyrrolidine-2-carbonitrile.

### 2.2. Experimental Animals

From the laboratory animal farm of the Faculty of Veterinary Medicine, Zagazig University, 50 male *Sprague-Dawley* rats (250 ± 5 g) were purchased. Each rat was sustained in a clean, pathogen-free environment between 21 and 24 °C, in a stainless-steel cage. The rats were each kept in a 12-h light-dark cycle with 60% relative humidity.

### 2.3. Preparation and Characterization of Liposomal BBR

#### 2.3.1. Preparation of Chitosan Modified BBR Loaded Nano-Liposomal Systems

Unmodified berberine-loaded liposomes were prepared by the ethanol injection method as previously reported [40,41]. Berberine (10 mg), hydrogenated soybean phosphatidylcholine (HSPC, 304 mg), and cholesterol (30.4 mg) were dissolved into 5 mL absolute ethanol and maintained at 60 °C to form the organic phase. The prepared organic solution was then injected into 10 mL of distilled water at 60 °C while stirring at 750 rpm using a syringe (25 G needle). The obtained suspension was kept at 60 °C for 30 min to allow for the evaporation of ethanol. Chitosan-modified liposomes (chitosomes) were prepared by adding 10 mL of 0.1 mL hydrochloric acid containing 0.2% chitosan solution dropwise to 10 mL of previously scheduled unmodified liposome suspension while swirling at 500 rpm at room temperature. After that, the mixture was continually stirred for an hour and stored at 4 °C until further investigation. The resulting berberine-loaded chitosomes had 20 mM phospholipids and 20% molar cholesterol. Encapsulation efficiency (%) was calculated as previously reported [42] via the lysis of liposomal systems and characterized for particle size, zeta potential, polydispersity index, and scanning electron microscopy.

#### 2.3.2. Vesicular Shape and Surface Morphology

Scanning electron microscopy (SEM) was used to characterize the surface and shape of liposomal preparations. A drop of liposomes was mounted in a glass stub, left to dry in air, covered with gold using a sputter coater, and visualized under a scanning electron microscope (JEM-1400, Jeol, Tokyo, Japan).

#### 2.3.3. Determination of Particle Size, Zeta Potential, and Polydispersity Index

A Malvern Zetasizer Nano (Malvern Instruments Ltd., Worcester, UK) was used to evaluate the particle size and polydispersity index (PDI) of the BBR-loaded chitosomes using dynamic light scattering (DLS). Particle-size testing samples were created by mixing 300 µL of the nanovesicle suspension with 3 mL of distilled water. At room temperature and a 173-degree detection angle, the particle sizes were assessed. The sample underwent three different tests. The level of homogeneity of the particle size is indicated by the PDI, which was developed as a measurement of the distribution of the nanovesicles population.

#### 2.3.4. Determination of Entrapment Efficiency

As previously described [42], BBR content is measured directly inside liposomal systems by lysing the systems with acetonitrile. Prior to and during the creation of BBR-Lip, free medication was isolated from liposomes using a centrifuge (Hermle^®^ Z326 K, Essen, Germany) by centrifuging 1 mL of liposomal solution at 15,000 rpm for 60 min at 4 °C. To ensure the complete removal of the un-entrapped medication, the separated liposomes were washed twice at two different phases by resuspending them in deionized water. The liposomes were then exposed to acetonitrile (1:4 liposome to acetonitrile) before being sonicated and vortexed to create a homogeneous solution. The level of BBR, encapsulated in liposomal systems, was then measured spectrophotometrically at 346 nm (Shimadzu UV-1800 UV/Visible, Kyoto, Japan) after the solution had been spun at 15,000 rpm for 30 min. Using equation 1, the entrapment efficiency (EE%) was estimated. The same techniques used to create BBR-free liposomes were used to prepare the blank.

(1) $$EE\%=\frac{Qs}{Qt}\times 100$$

where Qs is the amount of BBR found after the lysis of liposomes and Qt is the total amount of BBR added in the formulation of liposomes.

### 2.4. Experimental Induction of T2-DM

The high-fat-diet STZ model was utilized to induce type 2 diabetes using the previously outlined protocol [3]. The threshold at which stable hyperglycemia is seen in rats is 200 mg/dL.

### 2.5. Animals and Experimental Design

The rats were randomly allocated into five groups. The rats in the control non-diabetic group (10 rats) were given 0.5 mL/rat of normal saline orally via a feeding needle (intragastric route) for 14 weeks. The other 40 rats were used for the induction of T2DM via the HFD/STZ protocol described in detail in our previous study [3]. After becoming diabetic, they were divided into four equal groups. The first two groups are the T2DM group (the control diabetic group, given normal saline (0.5 mL per day) during the time of the experiment) and the T2DM-Lip-BBR group (via oral gavage at a dose equivalent to 10 mg/kg b.wt per day for 14 weeks) [43]. This dose is twice as high as the amount found to be optimal for an oral drug delivery protocol, when the authors compared the pharmacokinetic analysis of BBR-loaded chitosan-coated nano-liposomes to that of BBR-loaded uncoated nano-liposomes in rabbits and found that the BBR was more efficiently delivered orally via the chitosan-coated nano-liposomes. The last two groups are the T2DM-Vild group (diabetic rats were given vildagliptin (Vild) 10 mg/kg b.wt orally [44]) and the combined treatment group T2DM-Vild+ Lip-BBR, in which the diabetic rats were given Lip-BBR (10 mg/kg b.wt) and Vild (5 mg/kg b.wt), respectively, for 14 weeks.

### 2.6. Sampling

After the 10th week, all the rats were anesthetized by urethane (0.65 mL/100 g) after fasting for 12 h; The blood was drawn from the medial canthus into tubes without anticoagulants. The test tubes were centrifuged at 322× *g* for 10 min after being left at room temperature for 20 min. The serum was collected and frozen at −20 degrees Celsius until it was time to use it in biochemical tests. The final weights of the rats were recorded, and after euthanasia, the liver was dissected from each rat, and a 50 mg sample of each liver was rapidly disposed in 1 mL QIAzol (Qiagen, Hilden, Germany) and kept at −80 °C for gene expression analysis (RT-qPCR). Two grams of livers were homogenized (Ultra-Turrax homogenizer), and the homogenate was frozen at −20 degrees Celsius. Left lateral hepatic lobes were taken for histological and immunohistochemical analysis.

### 2.7. Biochemical Examinations

#### 2.7.1. Serum Glucose and Insulin Levels and HOMA-IR

Serum glucose was determined using the glucose oxidase technique with commercially available kits (ab219924, Cambridge, Cambridge, CB2, UK). An ELISA kit was used to quantify the insulin in the blood (RayBiotech, Peachtree Corners, GA, USA; Catalog No: ELR-Insulin). Insulin resistance was determined with HOMA-IR calculators [45].

#### 2.7.2. Lipid Profile

Serum TC, TAG, and HDL-c concentrations were measured using commercially available MyBioSource, San Diego, USA, kits and evaluated with an automatic biochemical analyzer (14,000 auto-analyzer, Toshiba, Tokyo, Japan). Friedewald’s formula was used to determine LDL-c [46].

#### 2.7.3. Liver Enzymes

Utilizing commercially available kits, the levels of alkaline phosphatase (ALP), aspartate aminotransferase (AST), and alanine aminotransaminase (ALT) in the serum were measured. Equipment was provided by Abcam (Cambridge, UK) (ab105134, ab105135, and ab8336, respectively) and used following the producer’s instructions.

#### 2.7.4. Hepatic Oxidants/Antioxidants Status

Following the manufacturer’s instructions, malondialdehyde (MDA) (MBS169313) was measured in liver homogenates. Superoxide dismutase (SOD) (MBS036924), catalase (MBS006963), and glutathione peroxidase (GPX) (MBS036923) levels were determined using ELISA kits (MyBioSource; San Diego, CA, USA) (CAT. NO. MBS028183) MDA.

#### 2.7.5. Estimation of Inflammatory Cytokines

Elisa assay kits of Komabiotech Inc. (Seoul, Republic of Korea) USA Catalog No. KBT-K0331196P, KBTK0331229, and KBT-K0331212 homogenate of hepatic tissue were employed to estimate TNF-, IL-6, and IL-10 levels, respectively, according to the manufacturer’s recommendations. Comparisons were made between the measured cytokine levels in the test samples and the concentrations of the standard curves generated from recombinant cytokines of standard solutions.

### 2.8. Real-Time Quantitative PCR (RT-qPCR) Analysis

The real-time analysis has been previously reported by Abd El-Hakim, et al. [47]. Initially, the total RNA was obtained from liver tissue using a Trizol Reagent (Thermo Fisher Scientific; Waltham, MA, USA). The NanoDrop^®^ ND-1000 Spectrophotometer was used to determine the concentration of the isolated RNA (NanoDrop Technologies, Wilmington, NC, USA). HiSenScriptTM RH (-) cDNA Synthesis Kits (iNtRON Biotechnology Co., Seongnam, Republic of Korea) were used in a Veriti 96-well thermal cycler to synthesize the first strand of cDNA (Applied Biosystems, Foster City, CA, USA). The primers’ sequences are listed in Table 1 according to the previously published articles of (Khamis et al., 2021) and [48]; the real-time PCR amplification was carried out according to the protocol of Livak and Schmittgen [49].

### 2.9. Histopathological Examination

The left lateral hepatic lobes of six rats per group were sampled and trimmed according to Ruehl-Fehlert, et al. [50] and fixed in a 10% neutral buffered formalin solution for 48 h. Post-fixation, representative hepatic tissue specimens were processed for paraffin impregnation and embedding [51]. Five-μm-thick tissue sections were obtained, stained with hematoxylin and eosin, and examined microscopically. Next, a multiparametric quantitative lesion scoring was established, where for each rat, five 40× and five different 10× nonoverlapped microscopic fields were snapshotted. After imaging, the 40× images were used to determine the proportions of hepatocytes that exhibited vacuolar and hydropic degenerations, apoptosis, or steatosis in relation to the total numbers of hepatocytes per image, and the 10× images were used to determine the frequencies of vascular congestions, leukocytic infiltrations, necrosis, hemorrhages, fibrosis, and cholestasis.

### 2.10. Immunohistochemical Staining and Quantitative Analysis of Autophagy Markers

Five-µm-thick sections of the formalin-fixed paraffin-embedded hepatic tissues were stained for Beclin-1 and LC3-II. Heat-mediated antigen retrieval was performed using citrate buffer, pH 6.0, and microwaved for 20 min. The sections were then incubated with the rabbit polyclonal anti-Beclin-1 primary antibody (ab62557) (Abcam, Cambridge, UK) at 1 µg/mL dilution and the rabbit monoclonal recombinant anti-LC3B primary antibody [EPR18709] (ab221794) (Abcam, Cambridge, UK) at 0.1 μg/mL dilution following the avidin-biotin-peroxidase complex technique [52]. The substance 3,3′-Diaminobenzidine was used as chromogen and hematoxylin as counterstain. Image J’s color deconvolution plugins were then used to quantify the immunoexpression of Beclin-1 and LC3-II in liver tissues in ten high-power microscopic fields per marker per rat [53].

### 2.11. Statistical Analysis

GraphPad INSTAT was used to perform the statistical analysis (Version 2). One-way analysis of variance (ANOVA) and Tukey’s multiple range test was used for the statistical analysis. Standard error and mean differences are presented. This analysis assigned significance to a *p*-value of 0.05 or lower.

## 3. Results

### 3.1. Characterization of Liposomal BERBERINE (Lip. BBR.)

**Figure 1:**
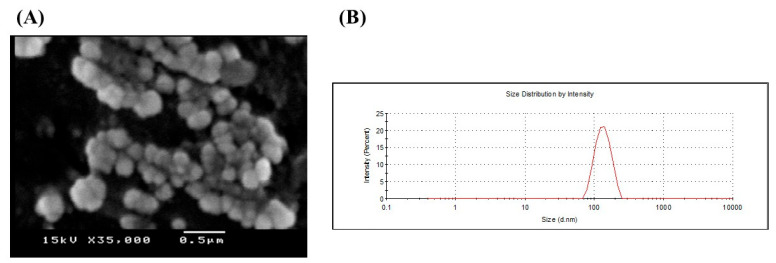
(**A**) Scanning electron microscope and (**B**) Size distribution chart of berberine hydrochloride loaded nano-liposomes (chitosomes). Berberine-loaded chitosomes were characterized for particle size and electron microscopy. SEM imaging revealed that liposomes are primarily spherical and demonstrate the three-dimensional structure of liposomes. Also, SEM confirmed the existence of liposomes in the nanosize with homogeneous size distribution. Liposomal average particle size was estimated via dynamic laser scattering (154 nm).

The results demonstrated that the chitosomes encapsulating berberine hydrochloride had a spherical form with a 3D character (Figure 1A). Additionally, the zeta potential of liposomes was +32.2 ± 1.53 mV. Their mean diameter was 154.2 ± 3.76 nm, with PDI equal to 0.214, indicating homogenous suspension (Figure 1B). The entrapment efficiency of the prepared modified liposomal systems was found to be low due to the hydrophilic nature of BBR hydrochloride (21.44% ± 3.55).

### 3.2. Effects of Lip-BBR on Body Weight in T2DM Rats

Table 2 displays data about the weight changes of the control group, the diabetic groups, and the groups treated with either monotherapy or a combination of Lip-BBR and Vild. There was no significant difference in the initial body weight among any experimental groups (*p* > 0.05). All rats fed with a high-fat diet for four weeks gained significantly more weight (*p* < 0.0001) than those given the control diet. Diabetic rats lost considerably (*p* < 0.001) more weight than control rats during the experiment, with the difference between the two groups being statistically significant at the 14-week point (*p* < 0.0001). On the other hand, when diabetic rats were co-treated with both Vild and Lip-BBR for 10 weeks, a significant (*p* < 0.0001) improvement in body weight was observed compared with the diabetic non-treated group. This seems substantial (*p* < 0.05) in comparison to the single-treated groups, demonstrating the efficacy of this approach in countering the weight loss often seen in diabetic rats.

### 3.3. Effects of Lip-BBR on the Levels of Fasting Blood Glucose, Serum Insulin, and Insulin Resistance (HOMA-IR)

As shown in Table 2, the fasting blood glucose levels of the induced T2DM rats were significantly (*p* < 0.0001) higher than those of the normal control rats. Oral supplementation of diabetic rats either by Lip-BBR or Vild, 10 mg/kg of each, significantly (*p* < 0.001) lowered the blood glucose levels of tested diabetic rats compared to the diabetic group. Interestingly, the combination of both treatments (Lip-BBR and Vild) in diabetic rats demonstrated significantly (*p* < 0.001) lowered glucose levels than the single-treated groups. The combination approach seems more effective in reducing the fasting blood glucose level and also assisted in minimizing the administered dose of vildagliptin to half the amount, which could be a possible benefit to replacing the chemicals with natural and healthier compounds.

Serum insulin levels were significantly (*p* < 0.0001) higher in the high-fat-diet-induced T2DM rats compared to the control rats, as shown in Table 2. The treatment of diabetic rats in the T2DM group with Lip-BBR (10 mg/kg b.wt) or Vild (10 mg/kg b.wt) for 14 weeks, on the other hand, significantly (*p* < 0.0001) reduced serum insulin levels in comparison to the diabetic non-treated group. Additionally, compared to the diabetic group, the combined treated group with both Vild (5 mg/kg b.wt) and Lip-BBR (10 mg/kg b.wt) significantly (*p* < 0.0001) showed reduced serum insulin levels. Like human diabetes, rat diabetes showed a statistically significant (*p* < 0.0001) elevation in HOMA-IR compared to normal control rats. In contrast, the HOMA-IR index was dramatically reduced in STZ-induced diabetic rats fed a high-fat diet with either a single treatment with Lip-BBR or Vild or a combination of the two.

### 3.4. Effects of Lip-BBR on Lipid Profile in T2DM Model

To find out whether Lip-BBR could ameliorate dyslipidemia in T2DM rats, the concentrations of TG, LDL-C, TC, and HDL-C were estimated in the serum of all groups. Compared with the control group, the TG and TC levels were significantly elevated in the diabetic model group. In contrast, the HDL-C level was distinctly decreased (*p* < 0.0001 Table 2), indicating that the induction of T2DM may initiate dyslipidemia. By comparison, Lip BBR and Vild treatment significantly (*p* < 0.001) lowered TG and TC levels and increased HDL-C levels (*p* < 0.001 and *p* < 0.0001). However, the combination of Lip-BBR and Vild potentially improved the serum lipid profile compared to T2DM-induced rats.

### 3.5. Effects of Lip-BBR on Hepatic MDA and Antioxidant Enzymes in T2DM Model

**Figure 2:**
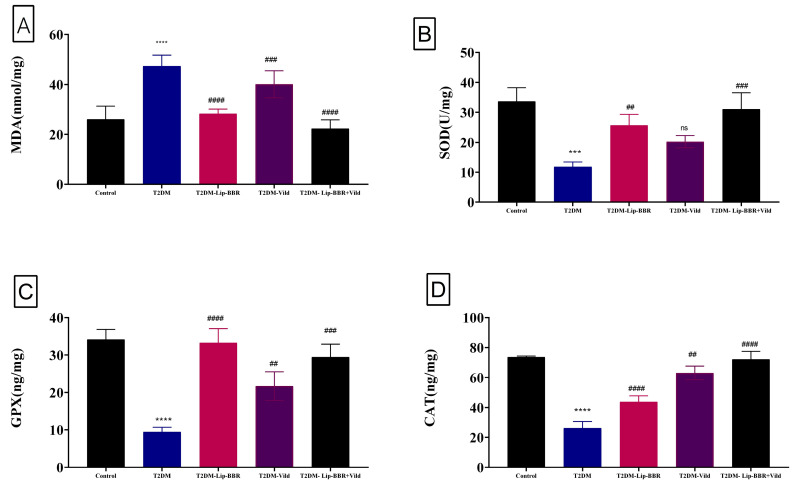
Effect of liposomal berberine (Lip-BBR) and/or vildagliptin (Vild) oral dosing for 14 weeks in diabetic male rats (T2DM) on hepatic levels of malonaldehyde (MDA) (**A**), superoxide dismutase (SOD) (**B**), glutathione peroxidase (GPX) (**C**), and catalase (CAT) (**D**) in the control and diabetic rats. Data expressed as mean ± SE, n = 6 for each group. The significance “****” is expressing the difference between the diabetic (T2DM group) and control (non-diabetic) *p* < 0.0001. The significance is expressing the difference among diabetic rats in the Lip. BBR and/or Vild vs. Diabetic (T2DM group) and “####” when *p* < 0.0001, “###” or “***” when *p* < 0.001, and “##” when *p* < 0.01, ns: no significance.

Figure 2 shows that hepatic MDA levels were nearly two-fold higher in diabetic rats than they were in control rats (*p* < 0.0001) (1.93 folds). The diabetic-Lip-BBR and Vild group had the lowest level of hepatic MDA, with about 58.65% less than the diabetic group, followed by the diabetic group administered Lip-BBR for 14 weeks after T2DM induction, indicating the effective role of Lip-BBR in protecting liver tissue from a high level of lipid peroxidation. Figure 2A also demonstrated that the group given Vild (10 mg/kg b.wt) showed significant (*p* < 0.001) determents in MDA level by 30.7% compared to T2DM rats. Regarding the antioxidant enzymes (SOD, GPX, and CAT) in Figure 2B–D, the induction of T2DM triggered significant decrements in these enzymes’ activity in the diabetic group by 64.92%, 72.36%, and 64.52%, respectively, relative to the control non-diabetic group. Furthermore, when the diabetic rats received Lip-BBR, the decrements were less pronounced compared to the diabetic group by 1.16 fold, 2.52 fold, and 67.57% for SOD, GPX, and CAT, respectively. Interestingly, when comparing the three diabetic groups, the co-treatment with Lip-BBR and Vild caused significant improvements in the activity of antioxidant enzymes (1.62 fold, 2.12 fold, and 1.76 fold increase, respectively) compared to non-treated diabetic rats.

### 3.6. Effects of Lip-BBR on Hepatic TNF-α, IL-10 & IL-6 Cytokines in T2DM

**Figure 3:**
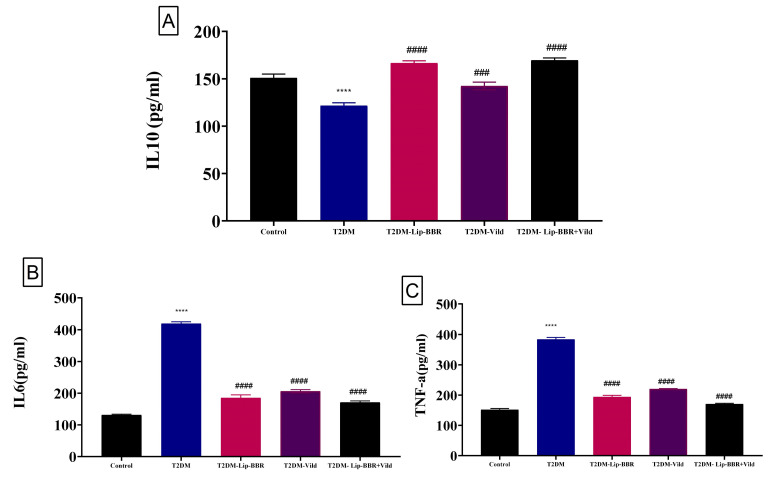
Effect of liposomal berberine (Lip-BBR) and/or vildagliptin (Vild) oral dosing for 14 weeks in diabetic male rats (T2DM). Data expressed as mean ± SE, n = 6 for each group on proinflammatory cytokines, including IL-10 (**A**), IL-6 (**B**), and tumor necrosis factor-α (TNF-α) (**C**), in hepatic tissue of control and diabetic rats. The significance “****” is expressing the difference between the diabetic (T2DM group) and control (non-diabetic), *p* < 0.0001. The significance is expressing the difference among diabetic rats in the Lip. BBR and/or Vild vs. Diabetic (T2DM group) and “####” when *p* < 0.0001, “###” when *p* < 0.001.

Data describing the effect of Lip-BBR on circulating proinflammatory cytokines in diabetic rats are summarized in Figure 3. Serum IL-10 showed a significant (*p* < 0.001) decrease following T2DM induction (19.47%). Treatment of the diabetic rats with Lip-BBR significantly (*p* < 0.001) elevated serum IL-10 levels by 37.01% compared with diabetic rats. The administration of Lip-BBR and Vild for 10 weeks for diabetic rats caused marked improvements in the IL-10 by 39.47% compared to diabetic rats. Also, T2DM caused a significant (*p* < 0.0001) increase in serum IL-6 and TNF-α levels by 2.19 fold and 1.52 fold, respectively, compared with the normal control rats. Oral supplementation of Lip-BBR could markedly (*p* < 0.0001) reduce the elevated serum levels of IL-6 and TNF-α when it is used as a single treatment by 55.83% and 49.54%, respectively, or used as a combined treatment with (Vild 5 mg/kg b.wt) by 59.26% and 55.56%, respectively (Figure 3B,C).

### 3.7. Effects of Lip-BBR on Hepatic Enzymes in T2DM

**Figure 4:**
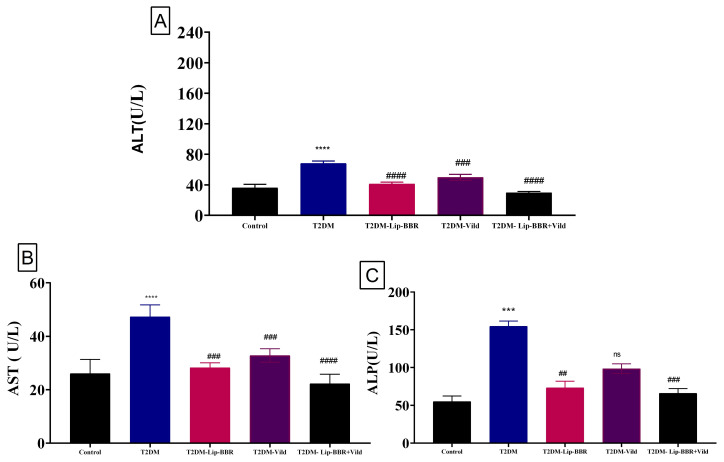
Effect of liposomal berberine (Lip-BBR) and/or vildagliptin (Vild) oral dosing for 14 weeks in diabetic male rats (T2DM). Data expressed as mean ± SE, n = 6 for each group on hepatocellular enzymes, alanine aminotransferase (ALT) (**A**), aspartate aminotransferase (AST) (**B**), and alkaline phosphatase (ALP) (**C**), in serum of control and diabetic rats. The significance “****” is expressing the difference between the diabetic (T2DM group) and control (non-diabetic), *p* < 0.0001. The significance is expressing the difference among diabetic rats in the Lip. BBR and/or Vild vs. Diabetic (T2DM group) and “####” when *p* < 0.0001, “###” or “***” when *p* < 0.001, and “##” when *p* < 0.01, ns: no significance.

Serum levels of ALT, AST, and ALP, sensitive indicators of liver function, are elevated after liver cells are damaged and the cell membrane becomes more permeable (Figure 4A–C). The serum levels of ALT, AST, and ALP in the diabetic group were significantly higher (*p* < 0.0001) by 88.95%, 81.64%, and 1.8 fold, respectively, compared with those in the control group, but this was reversed by the treatment with Lip-BBR even as a single treatment by 39.66%, 40.27%, and 81.7%, respectively. Moreover, the co-treatment of Lip-BBR and Vild caused too much restoration to nearly control values, considering a perfectly healthy state. This combination reduced the elevated levels of AST, ALT, and ALP by 56.48%, 52.88%, and 57.2%, respectively, compared to the diabetic group.

### 3.8. Lip-BBR Activates Autophagy in the Liver of T2DM Rats

**Figure 5:**
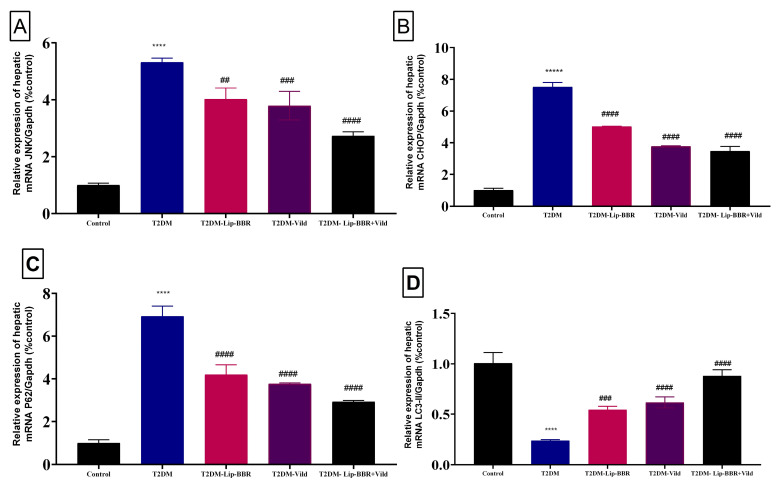
Effect of liposomal berberine (Lip-BBR) and/or vildagliptin (Vild) oral dosing for 14 weeks in diabetic male rats (T2DM). Data expressed as mean ± SE, n = 6 for each group on the relative expression of hepatic mRNA c-Jun N-terminal kinase (JNK) (**A**), C/EBP homologous protein (CHOP) (**B**), P62 (**C**), and light chain 3 phosphatidylethanolamine conjugate (LC3-II) (**D**) in control and diabetic rats. The significance “****” is expressing the difference between the diabetic (T2DM group) and control (non-diabetic) *p* < 0.0001. The significance is expressing the difference among diabetic rats in the Lip. BBR and/or Vild vs. Diabetic (T2DM group) and “####” when *p* < 0.0001, “###” when *p* < 0.001, and “##” when *p* < 0.01.

It is well established that autophagy and ERS work together functionally. In previous research, autophagy has been shown to improve dyslipidemia, oxidative stress, and inflammation and protect liver cells from injury and cell death [41,54]. Based on earlier research, the present study speculated autophagy. To scrutinize the effect of Lip-BBR on autophagy under the conditions of diabetic liver injury, liver autophagy and ERS were examined using gene expression and immunohistochemistry to detect the protein levels of Beclin1 and LC3-II in the liver. As presented in Figure 5A,B, the expressions of JNK and CHOP were significantly elevated (*p* < 0.0001) in the T2DM-induced group by HFD compared to the control non-diabetic group, indicating ERS in the liver tissue of diabetic rats. P62 has a role in proteasome degradation and is a potential autophagy activity indicator. It was found that P62 expression was elevated in the diabetic model group, but it was significantly (*p* < 0.0001) decreased after treatment with Lip-BBR as a single treatment or combined with Vild (*p* < 0.0001; Figure 5C).

**Figure 6:**
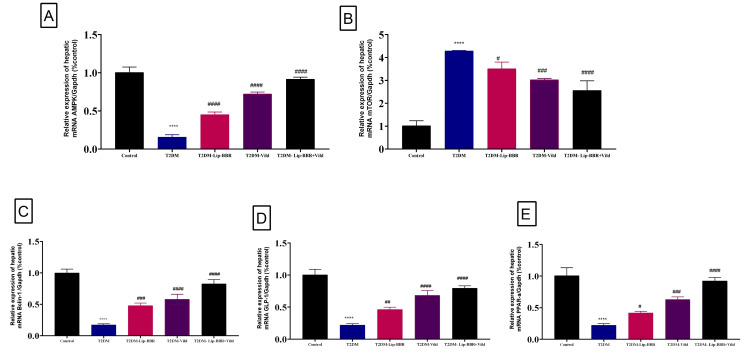
Effect of liposomal berberine (Lip-BBR) and/or vildagliptin (Vild) oral dosing for 14 weeks in diabetic male rats (T2DM). Data expressed as mean ± SE, n = 6 for each group on the relative expression of hepatic mRNA (AMPK) (**A**), mammalian target of rapamycin (mTOR) (**B**), Bclin-1 (**C**), glucagon-like peptide-1 (GLP-1) (**D**), and peroxisome proliferator-activated receptor alpha (PPAR-α) (**E**) in control and diabetic rats. The significance “****” is expressing the difference between the diabetic (T2DM group) and control (non-diabetic) *p* < 0.0001. The significance is expressing the difference among diabetic rats in the Lip. BBR and/or Vild vs. Diabetic (T2DM group) and “####” when *p* < 0.0001, “###” when *p* < 0.001, “##” when *p* < 0.01 and “#” when *p* < 0.05.

Moreover, there was upregulation in the expression pattern of LC3-II, a marker of autophagy [55], which was decreased in the diabetic group but was significantly (*p* < 0.0001) reversed by Lip-BBR treatment (Figure 5D). Beclin1 expression, a vital autophagic protein in the preliminary stages of autophagy, was downregulated in the diabetic group (*p* < 0.0001; Figure 6A). Still, it was significantly upregulated in the treatment of diabetic rats with Lip-BBR, and this was potentiated by the co-treatment with Lip-BBR and Vild treatment regarding the diabetic non-treated groups. mTOR, which prevents autophagy by blocking the development of lysosomal and autophagy-related protein complexes, controls cell growth and proliferation, keeps cellular energy homeostasis, and keeps mitochondria functioning normally. Figure 6B,C showed that the induction of T2DM caused an elevated expression of mTOR but inhibited GLP-1 compared to the control. Compared with the diabetic group, the treatment with Lip-BBR and/or Vild caused a substantial downregulation of mTOR and significant (*p* < 0.0001) upregulation of AMPK and GLP-1, but the effects were pronounced in the combination treatment group, which received Vild (5 mg/kg b.wt) concurrently with Lip-BBR. These results signify that Lip-BBR augments liver autophagy in T2DM rats via the AMPK/mTOR pathway.

Herein, gene expression analysis revealed a significant (*p* < 0.0001) downregulation of PPARα mRNA expression in hepatic tissue of the T2DM-induced diabetic rats when compared with the control group (Figure 6D). Alternatively, supplementation of the diabetic rats with either Lip-BBR alone or combined with Vild for 14 weeks produced a significant (*p* < 0.001) upregulation of hepatic tissue PPAR-α mRNA expression compared to the diabetic non-treated group.

### 3.9. Histopathology and Immunohistochemistry

**Figure 7:**
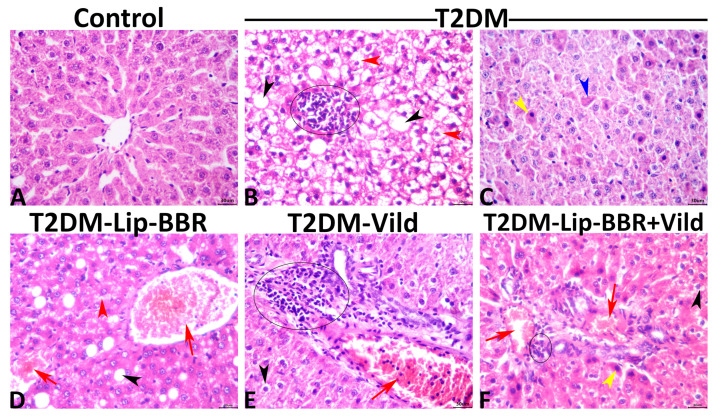
Representative photomicrograph of the hematoxylin and eosin-stained hepatic tissues shows normal histology in a control rat (**A**). The T2DM group shows microvesicular (red arrowheads), and macrovesicular (black arrowheads) steatosis with focal mononuclear cell aggregation (ellipse) (**B**), apoptotic hypereosinophilic hepatocytes with pyknotic nuclei (yellow arrowhead), or karyorrhectic nuclei (blue arrowhead) (**C**). The T2DM-Lip-BBR group shows vascular congestions (red arrows), microvesicular (red arrowhead), and macrovesicular steatosis (black arrowhead) (**D**). The T2DM-Vild group shows portal congestion (red arrow) and mononuclear infiltration (ellipse) (**E**). The T2DM-Lip-BBR + Vild group shows mild portal congestion (red arrows), few mononuclear infiltrations (ellipse), and a few apoptotic hepatocytes (yellow arrowhead) (**F**).

Normal histological pictures were seen in all livers of the control rats (Figure 7A), while a wide variety of pathologic changes were recorded in the hepatic tissue sections of the T2DM group. The primary lesions in all tissue sections included hepatic steatosis, which ranged from microvesicular steatosis (distended hepatocytes with foamy cytoplasm due to accumulation of small lipid droplets in the cytoplasm without nuclear displacement) to macrovesicular steatosis (large lipid droplets replacing all the cytoplasm with peripheral nuclear displacement). Frequently, this was associated with fusions of two or more hepatocytes besides mononuclear cell infiltration (Figure 7B). Another main lesion recorded in the majority of the hepatic tissue sections of this group was single-cell necrosis, which is predominantly caused by apoptosis, shrunken irregularly shaped hepatocytes with hypereosinophilic cytoplasm, and pyknotic, or karyorrhectic or karyolitic nuclei (Figure 7C). The other hepatic lesions noticed in this group included, but were not limited to, congestions of the sinusoids, central veins, portal blood vessels, vacuolar and hydropic degenerations, and focal necrotic areas usually infiltrated with inflammatory cell infiltrates. The biliary system was almost normal except for cholestatic changes in a few bile ducts. The hepatic tissue sections of the T2DM-Lip-BBR group manifested similar but milder lesions to those seen in the T2DM group. The basic alteration in all tissue sections of this group was hepatic steatosis associated with notable vascular congestion of the central and portal blood vessels (Figure 7D). Treatment with Vild showed significant hepatoprotective effects, as a marked reduction in the frequencies and extent of the diabetic-induced hepatic lesions were seen in the T2DM-Vild hepatic tissue sections. Mild to moderate histological alterations were still evident, including hepatocyte vacuolations, vascular congestions, and portal leukocytic infiltrations, particularly with mononuclear cells (Figure 7E). Concurrent treatment with Vild and Lip-BBR notably rescued the hepatic parenchyma from the diabetic-induced hepatopathic alterations, yet they did not regain the normal hepatic histology in the T2DM-Lip-BBR + Vild group. Few mild hepatopathic changes were seen in the group, including vacuolar and hydropic degenerations and portal congestion (Figure 7F). A quantitative lesion scoring for the hepatopathic alterations among all groups is summarized in Table 3.

### 3.10. Immunohistochemical Findings

**Figure 8:**
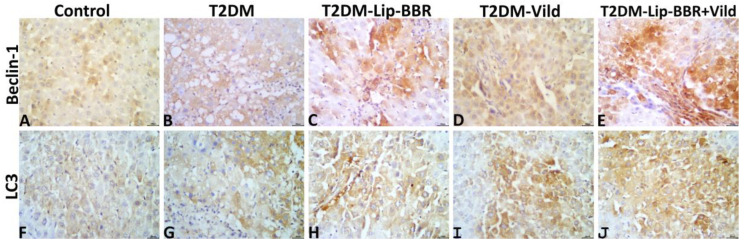
Representative photomicrograph of Beclin-1 stained (**A**–**E**) and LC3-II stained (**F**–**J**) hepatic tissue sections showing upregulation of both biomarkers’ immunoexpression in the T2DM group compared to the normal control group and notable upregulation of both biomarkers in the T2DM-Lip-BBR and T2DM-Vild groups, with higher upregulation in the T2DM-Lip-BBR + Vild group compared to the T2DM group.

The hepatic immunoexpression of Beclin-1 and LC3-II in all groups is shown in Figure 8A–J, respectively. Statistically, the data obtained from the image analysis declared that the immunoexpressions of the autophagic markers, Beclin-1 and LC3-II, were significantly higher in the hepatic tissue of the diabetic rats—T2DM, T2DM-Lip-BBR, T2DM-Vild, and T2DM-Lip-BBR + Vild—than in the hepatic tissue of the normal control rats. Treatment with either Lip-BBR or Vild significantly upregulated the Beclin-1 and LC3-II immunoexpression in the T2DM-Lip-BBR and T2DM-Vild groups compared to the T2DM. Interestingly, the higher upregulation of the Beclin-1 and LC3-II was recorded in the T2DM-Lip-BBR + Vild group, and there was no significant difference in the level of immunoexpression of both biomarkers between the T2DM-Lip-BBR and T2DM-Vild groups. For simplicity, the positively stained Beclin-1 and LC3-II brown area fractions in the hepatic tissue in ten high-power microscopic fields per rat among all groups are summarized in Table 3.

## 4. Discussion

The pharmacological properties of berberine (BBR), a quaternary isoquinoline alkaloid isolated from the roots, rhizomes, and stem bark of plants of the genus Berberis, include protection against neurodegeneration, cardiovascular disease, liver disease, high cholesterol, diabetes, and atherosclerosis [56]. Studies showed that less than 1% of orally administered BBR is totally accessible in rats [57]. Berberine’s limited oral bioavailability may result from its poor absorption and first-pass actions in the intestine and liver. Possible contributors to Berberine’s poor absorption include hepatobiliary re-excretion, P-glycoprotein-mediated efflux, limited permeability, and self-aggregation.

The current study observed that the T2DM-model group had vastly higher fasting blood glucose and insulin levels but significantly decreased body weight. Pathological alterations in the liver, such as hepatocyte abnormalities, lipid accumulation, and PPAR-α expression were also considerably increased in T2DM-rats. Also, there were elevated serum levels of ALT, AST, ALP, liver oxidative stress, and inflammation. However, Lip-BBR treatment alleviated the symptoms mentioned above, indicating that Lip-BBR had a potential role in alleviating diabetic liver injury in T2DM rats. Previous research has shown that BBR reduces blood glucose in both dietary and genetic rodent models of type 2 diabetes [58].

The present data indicates that the administration of Lip-BBR to diabetic rats caused an antihyperglycemic effect that could be due to BBR’s ability to regulate glucose metabolism, which may be the result of its impact on several different mechanisms and signal pathways, including but not limited to increasing insulin sensitivity; activating the adenosine monophosphate-(AMP-) activated protein kinase (AMPK) pathway; modulating gut microbiota; inhibiting liver gluconeogenesis; stimulating peripheral tissue cell glycolysis; and promoting intestinal glucagon-like protein-1 (GLP-1) secretion [59,60,61].

Herein, Lip-BBR alone or in combination with Vild significantly lowered insulin levels in diabetic rats when given for 10 weeks compared to the non-treated diabetic rats. Lip-BBR’s ability to safeguard islet function occurs via two mechanisms, which explains why it is so effective at causing this effect. First, BBR helped those with type 2 diabetes who also suffered from insulin resistance [62]. Additionally, in type 1 diabetes mellitus (T1DM) and the advanced stage of T2DM, where impaired islet function is present, BBR increases insulin secretion and preserves pancreatic islet cells via antioxidant activity [63]. In a study looking into the molecular mechanism of BBR against insulin resistance, Kong, et al. [64] found that BBR reduced fasting blood glucose (FBG) and fasting serum insulin by increasing insulin receptor (InsR) expression in in vitro and animal studies and by activating the promoter for InsR mRNA and protein production via protein kinase C (PKC) [65].

The etiology of diabetes mellitus (DM) and its consequences are linked to oxidative stress and inflammation [66,67]. By modulating oxidative stress indicators, antioxidant enzymes, and proinflammatory cytokines, recent research has shown that BBR can help modulate glucose homeostasis [68,69]. When given to diabetic rats in the current study, Lip-BBR (50 mg/kg b.wt) was shown to decrease malondialdehyde (MDA) and enhance superoxide dismutase (SOD), glutathione peroxidase (GPx), and catalase (CAT) enzyme, which was confirmed in several previous studies by [70,71]. All of these studies seek out the ability of BBR to scavenge free radicals and protect cells from oxidative stress, which is also efficiently done by Lip-BBR in our research. One study, however, found that this finding was debatable. The effect of BBR on oxidative stress was not apparent, as shown by the findings of Wang, et al. [72], who found no statistically significant difference between the MDA and SOD levels of diabetic mice and those of normal control animals (*p* > 0.05) after administration of BBR in non-nano form. Some previous studies declared the potential antioxidant role of BBR, including activation of SOD and GSH and its ability to decrease ROS production [66,73,74].

There is mounting evidence that combining BBR with oral hypoglycemics such as metformin, glipizide, or rosiglitazone improves glycemic control compared to either treatment method used alone. In contrast, the antidyslipidemic impact was more effective than oral hypoglycemic medications [56], whereas HDL-C, LDL-C, and TG showed no notable improvements. In our study, the combination of Lip-BBR with Vild showed a significant (*p* < 0.0001) reduction in the TC, TG, and LDLc compared to the diabetic rats.

In terms of mechanisms, it has been observed that berberine affects LDLR by post-translational modulation [75,76]. Berberine also decreases cholesterol in the blood by blocking the intestinal absorption, uptake, and release of cholesterol by enterocytes [77]. Since berberine reduces the expression of the acetyl-CoA acetyltransferase 2 gene, cholesterol is secreted less from enterocytes into the lymphatics. These findings are consistent with those seen in berberine-fed rats, which showed a 31% decrease in total plasma cholesterol, a 36% decrease in LDL-c, and a 45% decrease in the rate at which the body absorbed cholesterol from food [77]. This was also confirmed by the histopathological examination of liver tissue; Lip-BBR caused a marked reduction in microsteatosis, macrosteatosis, and hydropic degeneration. In this regard, Lip-BBR also caused upregulated expression of PPAR-α in the liver of diabetic rats treated with it, even as a single agent or combined with Vild. PPAR-α is a nuclear hormone receptor that controls fatty acid oxidation and transport [78]. Reversing steatosis in alcohol-fed animals using PPAR-α agonists has been demonstrated in mice [79].

The enzymes aspartate aminotransferase (AST) and alanine aminotransferase (ALT) are utilized as markers of liver health and function [40]. When hepatocytes are injured, their membranes become more permeable, leading to the release of the hepatic enzymes ALT and AST into the bloodstream. ALT and AST levels were considerably higher in the serum of the rats in the diabetic group compared with the normal animals. Following Lip-BBR administration, both groups’ ALT and AST levels dropped below those of the diabetic group. It is possible that Lip-BBR protects the liver against damage caused by oxidative stress, inflammation, and ER stress generated in different stages of type 2 diabetes. Diabetic rat livers were protected by the administration of Lip-BBR upon autophagy activation, glucose homeostasis, dyslipidemia, and antioxidant activation.

**Figure 9:**
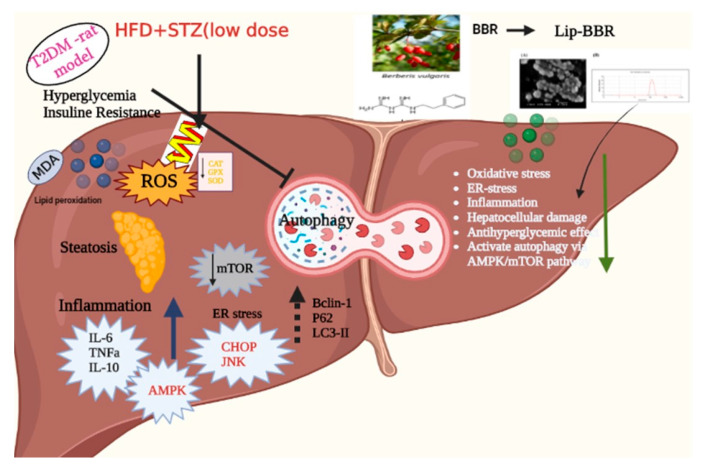
The major mechanistic pathways through which Lip-BBR could ameliorate the T2DM-induced hepatic tissue damage.

Additionally, based on these results, it is clear that Lip-BBR can stimulate liver autophagy in T2DM rats by influencing the AMPK/mTOR pathway. Autophagy is essential for cellular homeostasis as a self-repair mechanism [80]. Autophagy can be increased to help cells survive when AMPK is activated, but mTOR signaling is downregulated [81,82]. There is mounting evidence linking impaired autophagy function to diabetic liver damage [83,84]. Using quantitative real-time polymerase chain reaction (qRT-PCR), the current study found that liver autophagy ability was decreased in T2DM rats, as demonstrated by upregulated activity of P62 and m TOR and downregulated activity of LC3-II and Bclin-1 expression. This was additionally demonstrated by LC3-II and Bclin-1 immunohistochemistry staining in rat liver tissue samples. Consistent with prior research, these findings provide credence to autophagy’s function in the development of diabetic liver impairment in type 2 diabetes [83,84]. Restoring or stimulating autophagy protects the organs involved [85,86]. Similarly, the present research revealed that the autophagic activity in the liver of T2DM rats was restored after treatment with Lip-BBR, even with or without Vild but mostly better when the combined strategy was applied, which is promising for patients to avoid an overload of chemicals and drugs and receive healthy products, indicating that Lip-BBR reactivated the inhibited autophagy in the liver of T2DM rats (Figure 9). Also, autophagy has been shown to reduce dyslipidemia, oxidative stress, and inflammation [54,87]. These results suggest that autophagy plays a role in T2DM-related diabetic liver damage by reducing dyslipidemia, oxidative stress, and inflammation (Figure 9).

Lip-BBR was shown to reduce TNF- and IL-6 levels while increasing IL-10 and ameliorating the inflammatory state imposed on T2DM induction in rat liver tissue, as demonstrated by the current data. Lip-BBR has been shown to produce hypoglycemic effects, potentially by regulating inflammation [56], as shown in vitro and in vivo. Lip-BBR treatment can potentially reduce the production of inflammatory cytokines and acute-phase proteins. Protein folding and maturation occur in the ER lumen, offering a unique combination of molecular chaperones and folding enzymes. Recent research suggests that ERS and UPR signals are intertwined with hepatic lipid metabolism. In the current study, the expression of hepatic CHOP and JNK genes was found to be significantly elevated in diabetic rats when compared with that in control rats. Treatment with Lip-BBR significantly downregulated the expression of both CHOP and JNK in the liver of diabetic rats, which helps greatly in improving both the inflammatory state and hepatic cellular damage via ameliorating the rate of unfolded protein response and ER stress.

## 5. Conclusions

Liposomal encapsulation of berberine (Lip-BBR) plays a vital role in its oral administration, significantly enhancing its therapeutic effectiveness. In an animal model of type 2 diabetes, the intragastric delivery of Lip-BBR via liposomes exhibited remarkable protective effects against hepatic tissue damage, steatosis, inflammation, insulin resistance, and hepatocellular enzyme imbalance. Through the activation of autophagy in rat hepatocytes, Lip-BBR demonstrated its ability to shield hepatocytes from high glucose-induced damage, as evidenced by elevated levels of Bclin-1 and LC3-II proteins detected through immunohistochemical staining. Furthermore, when combined with Vild, a standard antidiabetic medication, Lip-BBR exhibited enhanced protective efficacy, allowing for a 50% reduction in dosage. These innovative findings not only offer insights into the molecular mechanisms underlying the hepatoprotective activity of Lip-BBR in diabetic liver damage but also hold promise for the development of novel therapeutics that modulate autophagy and ER stress.

## Figures and Tables

**Table 1:** Primer sequences, accession number, and product size for the quantitative RT-PCR for the analyzed genes in the hepatic tissue of control and diabetic rats.

Gene	Forward Primer (5′–3′)	Reverse Primer (5′–3′)	Accession No	Product Size
JNK	AGTGTAGAGTGGATGCATGA	ATGTGCTTCCTGTGGTTTAC	NM_053829.2	182
CHOP	CACAAGCACCTCCCAAAG	CCTGCTCCTTCTCCTTCAT	NM_001109986.1	158
P62	GGAAGCTGAAACATGGGCAC	CCAAGGGTCCACCTGAACAA	NM_181550.2	183
LC3-II	GAAATGGTCACCCCACGAGT	ACACAGTTTTCCCATGCCCA	NM_012823.2	147
Bclin-1	GAATGGAGGGGTCTAAGGCG	CTTCCTCCTGGCTCTCTCT	NM_001034117.1	180
mTOR	GCAATGGGCACGAGTTTGTT	AGTGTGTTCACCAGGCCAAA	NM_019906.2	94
GLP-1	CACCTCCTCTCAGCTCAGTC	CGTTCTCCTCCGTGTCTTGA	NM_012707.2	128
PPAR-α	GTCCTCTGGTTGTCCCCTTG	GTCAGTTCACAGGGAAGGCA	NM_013196.2	176
AMPK	GCGTGTGAAGATCGGACACT	TGCCACTTTATGGCCTGTCA	NM_023991.1	103

JNK: c-Jun N-terminal kinase LC3-II: light chain 3 phosphatidylethanolamine conjugate; CHOP: C/EBP homologous protein; mTOR : mammalian target of rapamycin; AMPK: MP-activated protein kinase; GLP-1: glucagon-like peptide-1; PPAR-α: peroxisome proliferator-activated receptor alpha.

**Table 2:** Effect of liposomal berberine (Lip. BBR) and/or vildagliptin (Vild) oral dosing for 14 weeks in diabetic male rats (T2DM). Data expressed as mean ± SE, n = 6 for each group on body weight at 0, 4, and 14 weeks, glucose level, insulin levels, HOMA IR, TC, TG, HDLc, and LDLc in serum of control and diabetic rats.

Estimated Parameters	Control	T2DM	T2DM-Lip-BBR	T2DM-Vild	T2DM-Lip-BBR + Vild
Initial Body weight (g)	183.7 ± 0.89	184.3 ± 0.33	181.7 ± 3.28	183.7 ± 2.03	184.3 ± 0.88
Body weight (g) at 4 weeks	218.3 ± 2.78	315.0 ± 2.89 ****	250.3 ± 2.91 ^####^	255.0 ± 0.58 ^####^	245.0 ± 2.89 ^####^
Body weight (g) at 14 weeks	305.0 ± 2.87	185.0 ± 2.88 ****	215.0 ± 2.59 ^###^	225.0 ± 2.46 ^###^	235.0 ± 2.66 ^####^
Glucose level (mmol/L)	4.00 ± 0.09	20.91 ± 0.62 ****	10.64 ± 0.33 ^####^	8.53 ± 0.33 ^####^	6.48 ± 0.32 ^###^
Insulin level	26.73 ± 1.25	55.21 ± 2.90 ****	32.09 ± 1.48 ^####^	30.90 ± 0.60 ^####^	29.07 ± 0.50 ^####^
HOMA IR	4.75 ± 0.27	51.17 ± 1.46 ****	15.13 ± 0.27 ^####^	11.71 ± 0.37 ^####^	9.710 ± 0.46 ^####^
TC (mg/dL)	115.5 ± 1.57	336.2 ± 10.82 ****	163.2 ± 6.52 ^####^	185.3 ± 3.20 ^####^	130.8 ± 9.19 ^####^
TG (mg/dL)	60.49 ± 1.93	233.9 ± 7.51 ****	125.2 ± 3.77 ^####^	163.3 ± 3.93 ^###^	105.7 ± 4.12 ^####^
HDLc (mg/dL)	38.45 ± 0.63	13.79 ± 2.08 ****	30.27 ± 0.57 ^###^	24.43 ± 1.71 ^##^	40.05 ± 2.26 ^####^
LDLc (mg/dL)	70.57 ± 2.37	200.6 ± 7.99 ****	82.26 ± 3.75 ^####^	129.0 ± 10.89 ^###^	79.85 ± 4.93 ^####^

The significance “****” is expressing the difference between the diabetic (T2DM group) and control (non-diabetic), *p* < 0.0001. The significance is expressing the difference among diabetic rats in the Lip. BBR and/or Vild vs. Diabetic (T2DM group) and “####”when *p* < 0.0001, “###” when *p* < 0.001, and “##” when *p* < 0.01.

**Table 3:** Quantitative lesion scoring and the immunoexpression of Beclin-1 and LC3-II in the hepatic tissue of different groups.

Lesion and Immunoexpression		Control	T2DM	T2DM-Lip-BBR	T2DM-Vild	T2DM-Lip-BBR + Vild
Beclin-1 (DAB area fraction)		5.76 ± 0.42	9.48 ± 0.6 ***	13.10 ± 0.47 ^###^	12.02 ± 0.56 ^##^	15.99 ± 0.32 ^####^
LC3-II (DAB area fraction)		4.10 ± 0.48	7.48 ± 0.61 ***	9.98 ± 0.40 ^##^	9.55 ± 0.45 ^#^	12.16 ± 0.44 ^###^
Congestions	Central veins	0.000 ± 0.000	31.67 ± 7.49 ***	21.67 ± 6.00 ^#^	15.00 ± 2.23 ^#^	11.67 ± 1.66 ^##^
	Portal blood vessels	0.000 ± 0.000	25.00 ± 6.14 ****	7.50 ± 4.77 ^####^	9.16 ± 2.38 ^####^	4.16 ± 0.83 ^####^
	Sinusoids	0.000 ± 0.000	15.00 ± 2.23 ****	8.33 ± 1.11 ^ns^	5.00 ± 2.24 ^##^	5.00 ± 2.23 ^##^
Vacuolar and hydropic degeneration		0.000 ± 0.000	28.56 ± 2.236 ****	14.25 ± 0.59 ^#####^	7.78 ± 0.76 ^#####^	4.73 ± 0.44 ^#####^
Microsteatosis		0.000 ± 0.000	9.93 ± 0.41 ****	8.29 ± 0.57 ^#####^	2.89 ± 0.66 ^#####^	1.89 ± 0.33 ^#####^
Macrosteatosis		0.000 ± 0.000	9.057 ± 0.44 ****	7.77 ± 0.59 ^#####^	3.07 ± 0.59 ^#####^	1.13 ± 0.28 ^#####^
Apoptosis		0.29 ± 0.05	4.40 ± 0.60 ****	2.26 ± 0.35 ^##^	1.17 ± 0.27 ^#####^	0.66 ± 0.29 ^#####^

The significance “****” is expressing the difference between the diabetic (T2DM group) and control (non-diabetic), *p* < 0.0001. The significance is expressing the difference among diabetic rats in the Lip. BBR and/or Vild vs. Diabetic (T2DM group) and “#####” when *p* < 0.00001, “####” when *p* < 0.0001, “###” when *p* < 0.001, and “##” or “***” when *p* < 0.01 and “#” when *p* < 0.05, ns: no significance.

## Data Availability

Not applicable.

## Abbreviations

T2DM, type 2 diabetes mellitus; HFD, high-fat diets; STZ, streptozotocin; FBG, fasting blood glucose; Vild, Vildagliptin; ALT, alanine aminotransferase; AST, aspartate aminotransferase; TC, total cholesterol; TG, triglyceride; HDL-C, high-density lipoprotein cholesterol; LDL-C, low-density lipoprotein cholesterol; HOMA-IR, homeostasis model assessment of insulin resistance; JNK, c-Jun N-terminal kinase. LC3, light chain 3 phosphatidylethanolamine conjugate; CHOP, C/EBP homologous protein; mTOR, mammalian target of rapamycin; AMPK, adenosine monophosphate-activated protein kinase; GLP-1, glucagon-like peptide-1; PPAR-α, peroxisome proliferator-activated receptor alpha.
